# Cationic Channel TRPV2 Overexpression Promotes Resistance to Cisplatin-Induced Apoptosis in Gastric Cancer Cells

**DOI:** 10.3389/fphar.2021.746628

**Published:** 2021-10-04

**Authors:** Simona Laurino, Pellegrino Mazzone, Vitalba Ruggieri, Pietro Zoppoli, Giovanni Calice, Antonella Lapenta, Mario Ciuffi, Orazio Ignomirelli, Giulia Vita, Alessandro Sgambato, Sabino Russi, Geppino Falco

**Affiliations:** ^1^ Laboratory of Preclinical and Translational Research, IRCCS-CROB Referral Cancer Center of Basilicata, Rionero in Vulture, Italy; ^2^ Biogem Scarl, Istituto di Ricerche Genetiche “Gaetano Salvatore”, Ariano Irpino, Italy; ^3^ UOC Clinical Pathology, Altamura Hospital, Altamura, Italy; ^4^ Trial Office, IRCCS-CROB Referral Cancer Center of Basilicata, Rionero in Vulture, Italy; ^5^ Endoscopy Unit, IRCCS-CROB Referral Cancer Center of Basilicata, Rionero in Vulture, Italy; ^6^ Pathology Unit, IRCCS-CROB Referral Cancer Center of Basilicata, Rionero in Vulture, Italy; ^7^ Department of Biology, University of Naples Federico II, Naples, Italy

**Keywords:** drug resistance, gastric cancer, TRPV2, calcium channels, target therapy

## Abstract

Gastric cancer (GC) is characterized by poor efficacy and modest clinical impact of current therapies, in which apoptosis evasion is relevant. Intracellular calcium homeostasis dysregulation is associated with apoptosis escaping, and aberrant expression of calcium regulator genes could promote GC drug resistance. Since we previously found a prognostic value for TRPV2 calcium channel expression in GC, we aimed to characterize the role of TRPV2 in cisplatin resistance. Using the TCGA-STAD dataset, we performed a differential gene expression analysis between GC samples in upper and lower tertiles of TRPV2 expression, and then through a gene set analysis, we highlighted the enriched ontology and canonical pathways. We used qRT-PCR to assess TRPV2 expression in three GC cell lines and flow cytometry to evaluate cisplatin-induced cell death rates. Calcium green-1-AM assay was used to estimate differences in intracellular Ca^2+^ concentrations after inhibition of TRPV2. We engineered AGS cell line to overexpress TRPV2 and used confocal microscopy to quantify its overexpression and localization and flow cytometry to evaluate their sensitivity to cisplatin. Consistent with our hypothesis, among enriched gene sets, we found a significant number of those involved in the regulation of apoptosis. Subsequently, we found an inverse correlation between TRPV2 expression and sensitivity to cisplatin in GC cell lines. Moreover, we demonstrated that inhibition of TRPV2 activity by tranilast blocks the efflux of Ca^2+^ ions and, in combination with cisplatin, induced a significant increase of apoptotic cells (*p* = 0.004). We also demonstrated that TRPV2 exogenous expression confers a drug-resistant phenotype, and that tranilast is able to revert this phenotype, restoring cisplatin sensitivity. Our findings consistently suggested that TRPV2 could be a potential target for overcoming cisplatin resistance by promoting apoptosis. Notably, our data are a prerequisite for the potential reposition of tranilast to the treatment of GC patients and anticipate the *in vivo* evaluation.

## Introduction

Gastric cancer (GC) was the sixth most commonly diagnosed cancer and the second cause of cancer-related deaths in 2018 worldwide (Bray et al., 2018). Since patients with early-stage GC are mostly asymptomatic and current screening methods are not effective, GC is frequently diagnosed at advanced and metastatic stages (Van Cutsem et al., 2016; Griffin-Sobel, 2017). Moreover, GC remains a disease characterized by high recurrence rates, poor prognosis, and limited clinical benefit for patients undergoing current therapeutic regimens (Van Cutsem et al., 2006; Cunningham et al., 2008; Koizumi et al., 2008; Bang et al., 2010; Jácome et al., 2016; Ilson, 2018). These unfavorable features reside in the marked inter- and intratumor histopathological and molecular heterogeneity (McGranahan and Swanton, 2015). These pieces of evidence suggest that treatment strategies must be tailored or personalized to each individual’s tumor features. Therefore, aided by the rise of high-throughput techniques, research efforts are ever more focused on deciphering the molecular nature of cancer biological hallmarks, such as cancer cell proliferation, migration, and apoptosis evasion (Hanahan and Weinberg, 2011).

Several mechanisms conferring to GC cells, a selective advantage to escape drug-induced apoptosis, have been described (Russi et al., 2019b). In this scenario, ion channels may play a role since they mediate several responses in cellular physiology and are often dysregulated in most types of cancer, including those of the gastrointestinal tract (Anderson et al., 2019; Ding et al., 2020; Stokłosa et al., 2020). Among the different types of ion channels, those involved in the regulation of Ca^2+^ flux have been found to affect the sensitivity of GC cells to apoptotic stimuli. Indeed, they prevent cytosolic Ca^2+^ overload by diminishing Ca^2+^ flux from the endoplasmic reticulum or by enhancing leakage of Ca^2+^ excess from cells (Kischel et al., 2019). In this view, we recently described, for the first time, a prognostic role for TRPV2 Ca^2+^ channel in GC patients that prompts further research on the biological mechanisms behind its function (Zoppoli et al., 2019). In brief, we found that higher TRPV2 expression levels were associated with shorter overall survival and that its expression increases with the GC pathological stage. Moreover, TRPV2 immunostaining tested negative in normal stomach mucosa and highly positive in GC tissue samples. On these bases, TRPV2 can represent a good candidate as a personalized therapeutic target for GC.

TRPV2 is a member of the vanilloid subfamily of transient receptor potential (TRP) channels, originally described for their role in sensory processes, but it is also responsible for cancer growth, metastasis, and chemoresistance through the dysregulation of calcium signaling (Santoni et al., 2020). TRPV2 is highly permeable to Ca^2+^ and is physiologically activated by heat above 52°C, osmolarity changes, and membrane stretch (Nabissi et al., 2010). It can regulate the ion flux in both directions, but the outward rectification current, the efflux of cations from the cell, is predominant (Caterina et al., 1999). TRPV2 is constitutively expressed in the central nervous system, but it is also found in non-neuronal tissues, suggesting a possible role in a broad repertoire of physiological functions, such as cell proliferation, differentiation, and apoptosis (Nabissi et al., 2010). Activation of TRPV2 or its overexpression was found to promote invasiveness of prostate cancer cells and is associated with advanced pathological stage in esophageal squamous cell carcinoma, respectively (Liberati et al., 2014b; Kudou et al., 2019).

In this work, we evaluated, *in vitro*, the effects of TRPV2 chemical inhibition on sensitivity to cisplatin-induced apoptosis. Firstly, we demonstrated a correlation between TRPV2 expression and resistance to cisplatin-induced apoptosis in three GC cancer cell lines. Then, we showed that TRPV2 acts by discharging Ca^2+^ ions from cells, and that its inhibition leads to an increase in intracellular Ca^2+^ levels. Thereafter, we observed that chemical inhibition of TRPV2 induces a significant increase of cisplatin-induced apoptosis, and overexpression of TRPV2 in a cisplatin-sensitive GC cell line confers a cisplatin-resistant phenotype. Overall, we highlight a possible role of TRPV2 as a personalized medicine target for the treatment of GC, likely in combination settings with current chemotherapeutics to enhance their efficacy.

## Materials and Methods

### Data Collection and Processing

The Cancer Genome Atlas (TCGA) (https://www.cancer.gov/about-nci/organization/ccg/research/structural-genomics/tcga) repository collected high throughput genomic and transcriptomic and clinical data of many tumors and their adjacent normal tissues from many organs. In this study, gene expression dataset and clinical data of stomach adenocarcinoma (STAD) samples were retrieved from our previous analysis (Russi et al., 2019a). We selected 192 samples fully annotated for histology, pathologic stage, and TNM:N. According to TRPV2 expression, we subgrouped 192 samples in “TRPV2 high,” those belonging to the top 33 percentile (tertile), and “TRPV2 low,” those falling in the lower 33 percentile.

### Differential Gene Expression and Gene Set Analyses

Differential expression analysis was performed using edgeR. We considered in the model the histology, the pathologic stage, and the TNM:N as confounding factors of the generalized linear model. A gene was considered as differentially expressed (DEG) between high and low TRPV2 samples if corrected (FDR) *p* value <0.05 and expression change was >1.5-fold (log_2_FC >0.58). Using the clusterProfiler package on these DEGs (Yu et al., 2012), we analyzed the functional profile of TRPV2 high samples to get the significantly enriched Ontology and Canonical Pathways gene sets (FDR adjusted *p* value <0.05).

### Cell Culture

AGS (RRID:CVCL_0139), KATO-III (RRID:CVCL_0371), and SNU-1 (RRID:CVCL_0099) GC cell lines were purchased from ATCC (Manassas, VA, United States) and were cultured in their specific complete medium according to manufacturer instructions. GC cells were incubated at 37°C in 5% CO_2_; medium was changed daily, and cells were split every 2-3 days routinely.

### Apoptosis Assay for the Evaluation of Cisplatin-Induced Apoptosis

GC cell lines were exposed to different concentrations of cisplatin (0–40 µM) (Accord Healthcare Limited, Harrow, United Kingdom) for 48 h to assess their sensitivity. Similarly, to evaluate the effect of TRPV2 blockade by tranilast (Tocris Bioscience, Bristol, United Kingdom) on cisplatin resistance of KATO-III cells, cisplatin (10 µM) was added to cell culture after 10 min since the addition of TRPV2 inhibitor (250 µM). After 48 h of culture, KATO-III cells were recovered by trypsinization and subjected to flow cytometry. In brief, cells were washed twice with cold PBS and then, after centrifugation, resuspended in 100 *µ*L of 1x binding buffer at a concentration of 1 × 10^6^ cells/ml. Subsequently, 5 *µ*L of FITC Annexin V and 5 *µ*L propidium iodide (PI) (BD Biosciences, San Jose, CA, United States) were added, and cells were incubated for 15 min at room temperature (RT) in the dark. For each tube, an adequate volume of 1x binding buffer was added. All samples were acquired, within 1 h, by using a NAVIOS flow cytometer and analyzed by Kaluza software (Beckman Coulter Diagnostics, Brea, CA, United States). A total of 10^4^ events were acquired for each sample. Data from treated samples were normalized as fold change of their untreated controls and were reported as mean ± SE of at least three independent experiments.

### Evaluation of TRPV2 Gene Expression by qRT-PCR

For qRT-PCR analysis of TRPV2 expression in GC cells, total RNAs were collected immediately after cells recovering by TRIzol (ThermoFisher Scientific, Waltham, MA United States) according to the manufacturer’s instructions. One microgram of total RNA was reverse-transcribed by the transcriptor first-strand cDNA synthesis kit (Roche Life Science, Mannheim, Germany) according to the manufacturer’s instructions. qRT-PCR analyses were performed using 10 ng cDNA per well in triplicate with LightCycler^®^ 480 SYBR Green I Master (Roche Life Science) on LightCycler 480 Instrument (Roche Life Science). TRPV2 expression levels were estimated by normalizing C_t_s with three different housekeeping genes or their mean: DEDD, EEF1A1, and GAPDH. In amplification reactions, we used the following primers: Hs_TRPV2_1_SG QuantiTect Primer Assay (Qiagen, Hilden, Germany), DEDD-Fw-TCAGATGTGTAGCAAGCGGC, DEDD-Rev-AGTATTCAGCCCG AACCCG, EEF1A1-Fw-GCTCTGGACTGCATCCTACC, EEF1A1-Rev-GAACACCAGTCTCCA CTCGG, GAPDH-Fw-CCTCTGACTTCAACAGCGACA, and GAPDH-Rev-GCTGTAGCCAAAT TCGTTGTC. Cycling conditions were those from the datasheet, and annealing temperature has been set at 58°C for all primers pairs.

### Fluorescent Staining to Evaluate Free Intracellular Ca^2+^ Variation After TRPV2 Chemical Inhibition

KATO-III cells were cultured as described above in the presence of fluorescent calcium indicator calcium green-1 AM (ThermoFisher Scientific) at a final concentration of 5 *µ*M and incubated in the dark at 37°C. After 15 min of loading, different concentrations (10, 50, and 250 *µ*M) of the TRPV2 specific inhibitor tranilast were added and incubated at 37°C for 30 min. Subsequently, the 6-well plates were observed under a fluorescence microscope with a ×20 objective (Leica SP8 confocal microscope, Wetzlar, Germany) to evaluate baseline fluorescence intensity. Then, ionomycin (ThermoFisher Scientific) at 5 µM was added to induce elevation of intracellular Ca^2+^ levels, and fluorescence was monitored for 5 min. We used dimethyl sulfoxide (DMSO) (Sigma-Aldrich, St. Louis, MO, United States) as control since it was the vehicle of tranilast, calcium green-1 AM, and ionomycin. To quantify the differences in fluorescence intensities between samples, we carried out flow cytometry evaluation. In brief, cells were cultured in the same conditions, and events were acquired for 30 s to obtain baseline values and for 5 min after ionomycin exposure. Ionomycin-dependent intracellular Ca^2+^ concentration increase, after TRPV2 inhibition, was expressed as percent increase of mean fluorescence intensity as compared with untreated control. All data were obtained from three different experiments.

### Western Blot Analyses

Total proteins were extracted with RIPA buffer and 1x protease inhibitor cocktail (ThermoFisher Scientific). Protein concentration was determined using the Bio-Rad protein assay kit according to the manufacturer’s instructions. Forty micrograms of protein were separated on SDS-PAGE and transferred onto nitrocellulose membranes. These were incubated overnight at 4°C with the following primary antibodies, according to the different experiments: rabbit anti-human-TRPV2 (1:500, Sigma-Aldrich), rabbit anti-human PARP and cleaved-PARP (1:1000, Cell Signaling Technology, Leiden, Netherlands), rabbit anti-human CASP3 (1:1000, Cell Signaling Technology), and rabbit anti-human ACTB (1:5000, Cell Signaling Technology). After thorough PBS washing, blots were incubated with HRP-conjugated mouse anti-rabbit IgG (1:5000, Cell Signaling Technology). Protein bands were revealed by Clarity Western ECL Substrate and ChemiDoc System (Bio-Rad Laboratories, Berkeley, CA, United States). Densitometry analyses on data from three independent replicates were performed with ImageJ.

### Generation of a GC Cell Line Stably Overexpressing the TRPV2 Gene

To generate TRPV2-pIRES-Luc construct, luciferase coding sequence (CDS) was amplified by PCR from the PGL3-basic vector (Promega Corporation, Madison, WI, United States) and inserted in SalI/NotI (New England Biolabs, Ipswich, MA, United States) sites of the pIRES vector (Takara Bio United States, Mountain View, CA, United States). Subsequently, TRPV2 CDS was amplified by PCR from TRPV2-pCDNA3 (Genscript, Piscataway, NJ, United States), and it was inserted in EcoRI/MluI (New England Biolabs) sites of the pIRES-Luc vector. The construct was verified by restriction analysis and sequencing. Stably transfected AGS cells were generated by transfection of SpeI (New England Biolabs) linearized TRPV2-pIRES-Luc construct into wild-type AGS cells using Lipofectamine 2000 (ThermoFisher Scientific). After 48 h since transfection, positive clones were selected for Neomycin resistance. After 2 weeks of G418 (ThermoFisher Scientific) treatment, neoresistant (NeoR) clones were picked, propagated, and tested for TRPV2 expression by ddPCR and confocal microscopy.

### Droplet Digital PCR for TRPV2 Expression Quantification in Engineered Cells

About 10 ng of cDNA generated from 1000 ng of total RNA reverse transcribed with the transcriptor first-strand cDNA synthesis kit (Roche) was used. Droplet digital PCR (ddPCR) was performed using the QX200™ Droplet Digital™ PCR system (Bio-Rad Laboratories) including the droplet generator and the reader. Reactions were prepared in 20 µL volumes with QX200™ ddPCR™ EvaGreen Supermix (Bio-Rad Laboratories). As above, we used Hs_TRPV2_1_SG QuantiTect Primer Assay (Qiagen) and DEDD as a housekeeping gene. After droplet generation in DG8TM Cartridges (Bio-Rad Laboratories), ddPCR™ 96-well PCR plates containing reactions in duplex were loaded onto Veriti 96-well Thermal Cycler (Applied Biosystems) and cycled as follows: 5 min at 95°C, 40x (30 s at 95°C and 60 s at 58°C), 5 min at 4°C, 5 min at 90°C, and held at 4°C. QuantaSoft Software v1.7 (Bio-Rad Laboratories) was used to analyze the output of QX200™ Droplet Reader (Bio-Rad Laboratories). Relative TRPV2 expression was calculated as ratio of copies/µL as follows:

$$FC=\frac{{\left(TRPV2/DEDD\right)}_{poolTRPV2}}{{\left(TRPV2/DEDD\right)}_{eVect}}.$$

### Fluorescent Staining for Confocal Microscopy

About 3 × 10^4^ cells/well grown for 24 h on 2.13 cm^2^ 4-well chamber slides (SPL Lifesciences, Pocheon, South Korea) were fixed for 30 min at RT with 4% paraformaldehyde (pH 7.4) followed by 10 min at 37°C, washed three times with PBS, and then permeabilized for 10 min with 0.1% Triton X-100 in PBS. Blocking was performed by using a 0.5% solution of BSA in PBS at RT for 15 min. Cells were then incubated overnight at 4°C with rabbit anti-human TRPV2 primary antibody (Sigma-Aldrich). After three PBS washings, cells were incubated with the Alexa Fluor 488-Goat Anti-Rabbit IgG secondary antibody (ThermoFisher Scientific) for 1 h at RT. Slides were washed three times with PBS and mounted using ProLong™ Glass Antifade Mountant with NucBlue™ (ThermoFisher Scientific). Imaging was performed by using a Leica SP8 confocal microscope (Leica Microsystems) with a ×63 oil objective, exciting stained cells with 488 nm laser light.

### Statistical Analyses

Data were reported as counts, normalized counts, or fold changes as mean ± SE or median with range, as appropriate. Data were transformed to fit normal distribution as necessary. Differences among groups were evaluated by one-sample *t*-test, *t*-test, and ANOVA with Tukey’s post hoc test according to the nature of data. *p* values <0.05 were considered significant. Analyses were performed using RStudio and R software (RStudio, Inc., 2019; R Foundation for Statistical Computing, 2014).

## Results

### Biological Features of GC Samples with High TRPV2 Expression

**FIGURE 1:**
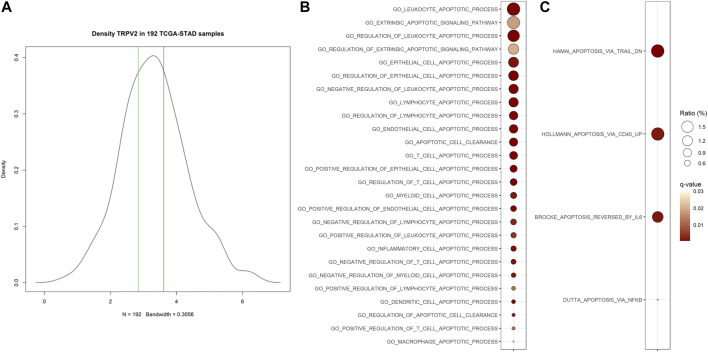
Association of TRPV2 expression with apoptosis-related gene sets. **(A)** Distribution on TCGA-STAD GC samples based on TRPV2 expression. Green and red lines define the lower and upper tertiles, respectively. **(B)** and **(C)** Apoptosis-related Ontology and Canonical Pathways gene sets significantly enriched in samples characterized by high TRPV2 expression. Ratio indicates, among all DEGs, the percent of upregulated genes included in each gene set.

To evaluate the biological features associated with high expression levels of TRPV2 gene, we performed a differential gene expression analysis between GC samples from the TCGA-STAD project falling in the upper tertile *versus* those in the lower one of TRPV2 expression values distribution (Figure 1A). Thereafter, using the 2,909 differential genes, we performed a gene set analysis (GSA) to highlight the biology of samples with high TRPV2 expression. We considered the Ontology (GO) and Canonical Pathways (CP) gene sets, finding 1,183 GO and 1,089 CP significantly enriched (Supplementary Material). Since we hypothesize a link between TRPV2 expression/activity and chemoresistance through calcium-mediated apoptosis regulation, we focused on those gene sets with “APOP” in their name. We found significantly enriched 26 out of 156 and 4 out of 42 apoptosis-related GO and CP, respectively (Figures 1B,C). According to Fisher’s test, such ratios of “APOP” gene sets result higher than expected by chance in GO. Interestingly, in line with our hypothesis, the top GOs include genes with antiapoptotic activity, such as BCL2, IGF1, and SERPINE1. Based on these findings, we sought to characterize in *in vitro* the possible mechanism by which TRPV2 can influence the apoptotic process.

### Basal TRPV2 Expression Levels in GC Cell Lines Are Associated with Resistance to Cisplatin-Induced Apoptosis

**FIGURE 2:**
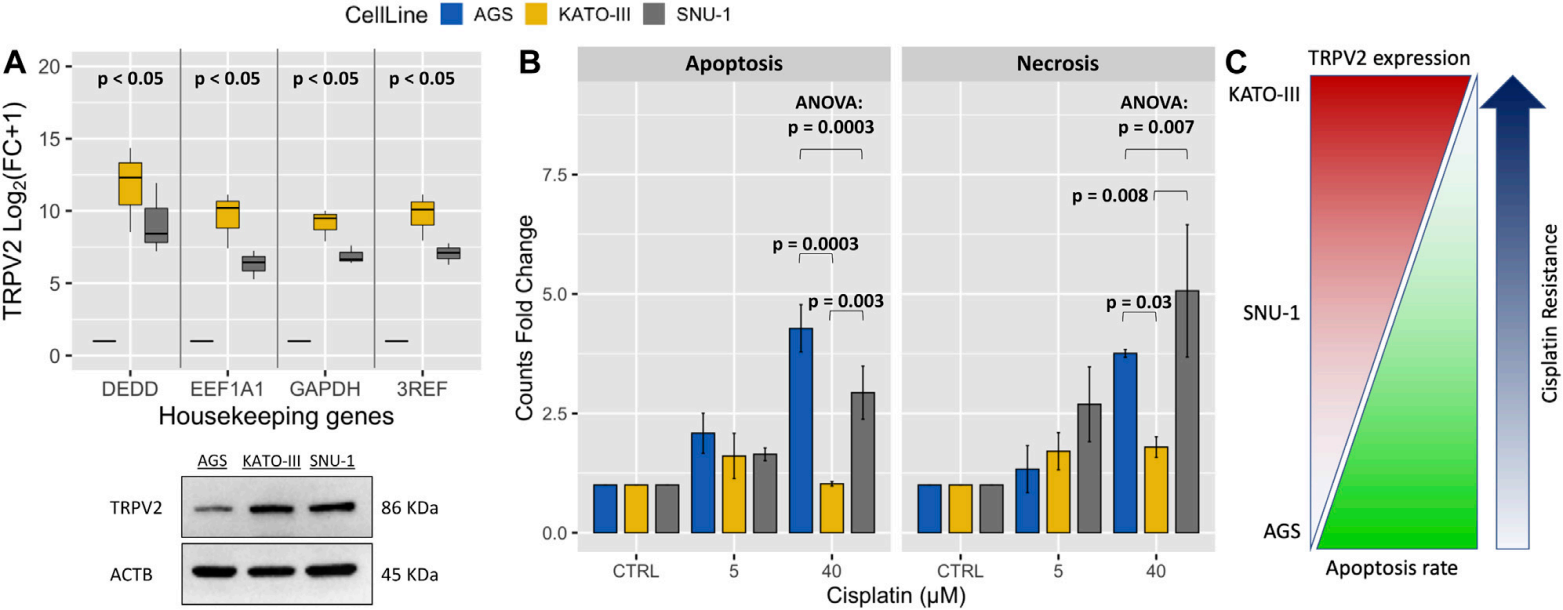
Association of TRPV2 expression with resistance to cisplatin-induced cell death in GC cell lines. **(A)** Relative qRT-PCR quantification of TRPV2 mRNA in GC cell lines by using three reference genes. TRPV2 expression is reported as FC relative to that in the AGS cell line. In the lower panel, western blot analysis of TRPV2 expression in the three GC cell lines. **(B)** Assessment of apoptosis and necrosis rates in several GC cell lines after treatment with different concentrations of cisplatin. Statistical significance was estimated by ANOVA test and Tukey’s post hoc test on three replicates. **(C)** Schematic representation of correlation between TRPV2 expression and cisplatin resistance of different GC cell lines. 3REF: mean of fold change (FC) from the different housekeeping genes.

To assess whether TRPV2 expression can influence the sensitivity of GC cell lines to drug-induced apoptosis, we first measured its expression levels by both qRT-PCR and western blot in three GC cell lines. By one sample *t*-test on log_2_(FC+1) values, we found that TRPV2 expression was significantly higher in both KATO-III and SNU-1 cell lines as compared with AGS one. This was also confirmed by western blot (Figure 2A). Interestingly, these results reflected the sensitivity of these GC cell lines to cisplatin-induced apoptosis assessed by flow cytometry. Indeed, KATO-III cells showed the least amount of cell death as compared with the other cell lines that die for both apoptosis and necrosis (Figure 2B). These results are consistent with data from Genomics of Drug Sensitivity in Cancer Project that reports a 60-fold higher cisplatin IC50 for the KATO-III cell line as compared to the other two cell lines (612.3 vs. 10.6 µM and 10.8 µM) (Yang et al., 2013). As outlined in Figure 2C, we found a proportional increase of GC cell line’s resistance to cisplatin with higher levels of TRPV2 expression.

### TRPV2 Modulates Intracellular Ca^2+^ Levels Through the Efflux of Ions from Cells

**FIGURE 3:**
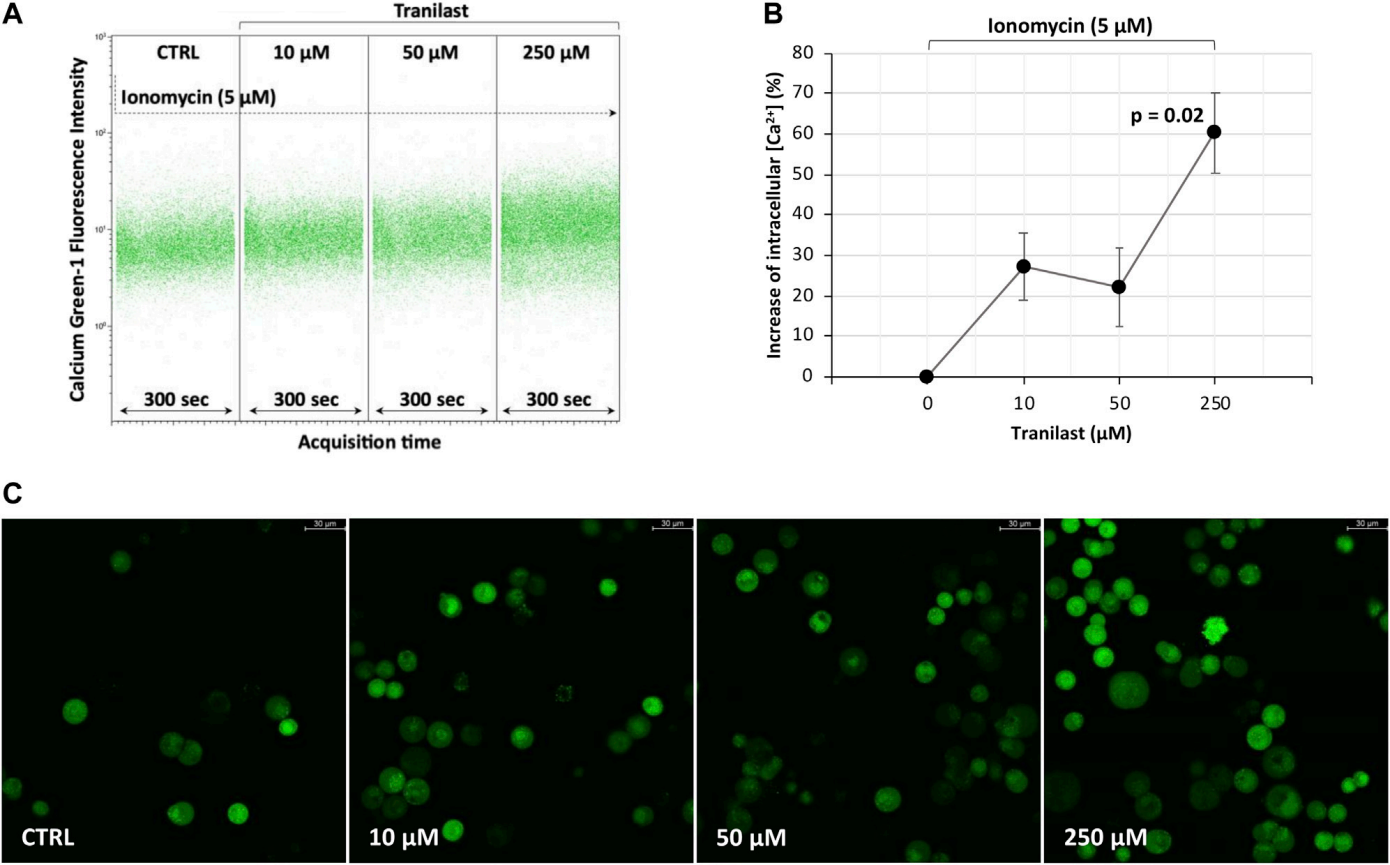
Effect of TRPV2 chemical inhibition on intracellular calcium levels. **(A)** Representative flow cytometry dot plots of calcium green-1-AM staining to measure intracellular calcium levels. **(B)** Estimation of mean fluorescence intensity differences measured by flow cytometry among the tranilast-treated groups and control. *t*-test was used to perform multiple comparisons on three replicates. **(C)** Fluorescence microscopy evaluation of intracellular calcium levels in the presence of ionomycin (5 µM) and different concentrations of TRPV2 inhibitor (×20 magnification, Leica SP8 microscope).

To evaluate how the activity of the TRPV2 channel influences intracellular calcium levels, we treated KATO-III cells with different concentrations of the specific TRPV2 inhibitor, tranilast, in the presence of ionomycin, an effective Ca^2+^ ionophore that is commonly used to modify intracellular Ca^2+^ concentrations. According to the previously described outward rectification current of the TRPV2 channel (Caterina et al., 1999), a 60.3% ± 9.8 mean increase of intracellular Ca^2+^ concentration was measured by flow cytometry when using 250 µM of tranilast as compared with untreated control in the presence of ionomycin (*p* = 0.02). On the contrary, for lower doses of tranilast, ionomycin exposure did not affect intracellular Ca^2+^ concentration, being the mean increase of 27.2% ± 8.2 (10 µM) and 22.1 ± 9.7 (50 µM) not significant (Figures 3A,B). A similar picture was observed under fluorescent microscopy (Figure 3C). Therefore, we hypothesize that, in KATO-III cells, TRPV2 maintains intracellular calcium concentration at low levels by favoring the leakage of Ca^2+^ ions.

### TRPV2 Chemical Inhibition Reverts Resistance to Cisplatin-Induced Apoptosis of KATO-III Cells

**FIGURE 4:**
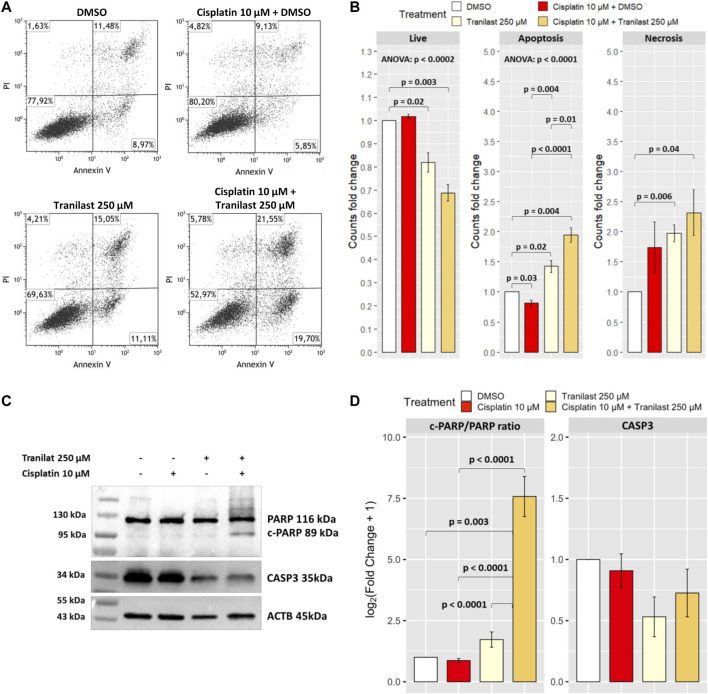
Effect of TRPV2 chemical inhibition on apoptosis rates of KATO-III cells. **(A)** Representative dot plots obtained by flow cytometry measurement of Annexin V exposure and propidium iodide (PI) fluorescence in different culture conditions. **(B)** Statistical analysis on DMSO normalized counts (fold changes) of the different types of cell death by one-sample *t*-test, ANOVA, and Tukey’s post hoc tests on three replicates. **(C)** Representative western blot of apoptosis activation markers in KATO-III cells treated with cisplatin, tranilast, or their combination. **(D)** Statistical analysis on log_2_-transformed actin-normalized densitometry data of common apoptosis markers amounts relative to cells treated with vehicle. ANOVA and Tukey’s tests were used to assess differences among means of treated cells, whereas one-sample *t*-test was used for comparison with the vehicle. Data were obtained from triplicates.

Assuming that TRPV2 contributes to evasion from cisplatin-induced apoptosis by lowering intracellular Ca^2+^ levels, we evaluated whether its inhibition can overcome this mechanism of drug resistance. Thus, we cultured KATO-III cells in the presence of cisplatin (10 µM) and tranilast (250 µM) alone or in combination. After 48 h, by flow cytometry, we measured the amounts of apoptotic and necrotic cells in the three different culture conditions compared with cells in the presence of the vehicle (DMSO). We observed a significant reduction of live cells after the exposure to tranilast alone and much more when tranilast was combined with cisplatin (61.9% ± 5.2 and 52.0 ± 4.1 *versus* 75.2% ± 2.8). More in detail, among the different types of cell death, this drug combination mainly determined an increase of apoptosis as compared with cisplatin alone (35.2% ± 2.1 *versus* 14.8% ± 0.6) (*p* < 0.0001) (Figures 4A,B). Conversely, cisplatin alone did not significantly induce cell death in KATO-III cells, but rather we observed a reduction of apoptosis rates (annexin V and propidium iodide-stained cells) as compared with vehicle-treated cells (14.8% ± 0.6 *versus* 18.3% ± 1.2) (*p* = 0.03). Single treatment with tranilast, instead, determined a significant increase of apoptosis compared with cisplatin alone (25.7% ± 0.7 *versus* 14.8% ± 0.6) (*p* = 0.004) but with lesser extent as compared with the combination of the two drugs (35.2% ± 2.1) (*p* = 0.01). Regarding necrotic cell death (propidium iodide stained cells), we did not find an increase of necrosis after the exposure to tranilast alone (12.3% ± 4.6) and in combination with cisplatin (12.8% ± 3.5) as compared with cisplatin alone (8.6% ± 1.8).

We confirmed the activation of apoptosis pathway by western blot analysis of common apoptosis markers. As for flow cytometry results, we observed a significant increase of the apoptosis marker cleaved-PARP only in KATO-III cells treated with the combination of cisplatin and tranilast as compared with untreated cells (*p* = 0.003) or cells treated with cisplatin (*p* < 0.0001) and tranilast (*p* < 0.0001) alone. Moreover, although not significant, a concomitant 50 and 30% reduction of CASP3 was reported for tranilast alone and its combination with cisplatin, respectively (Figures 4C,D). These findings support our hypothesis for a role of TRPV2 in resistance to cisplatin-induced apoptosis and demonstrate that tranilast is capable of unlocking apoptosis pathway by inhibiting TRPV2-mediated calcium leakage.

### TRPV2 Overexpression Confers a Cisplatin-Induced Apoptosis Resistant Phenotype to AGS Cells

**FIGURE 5:**
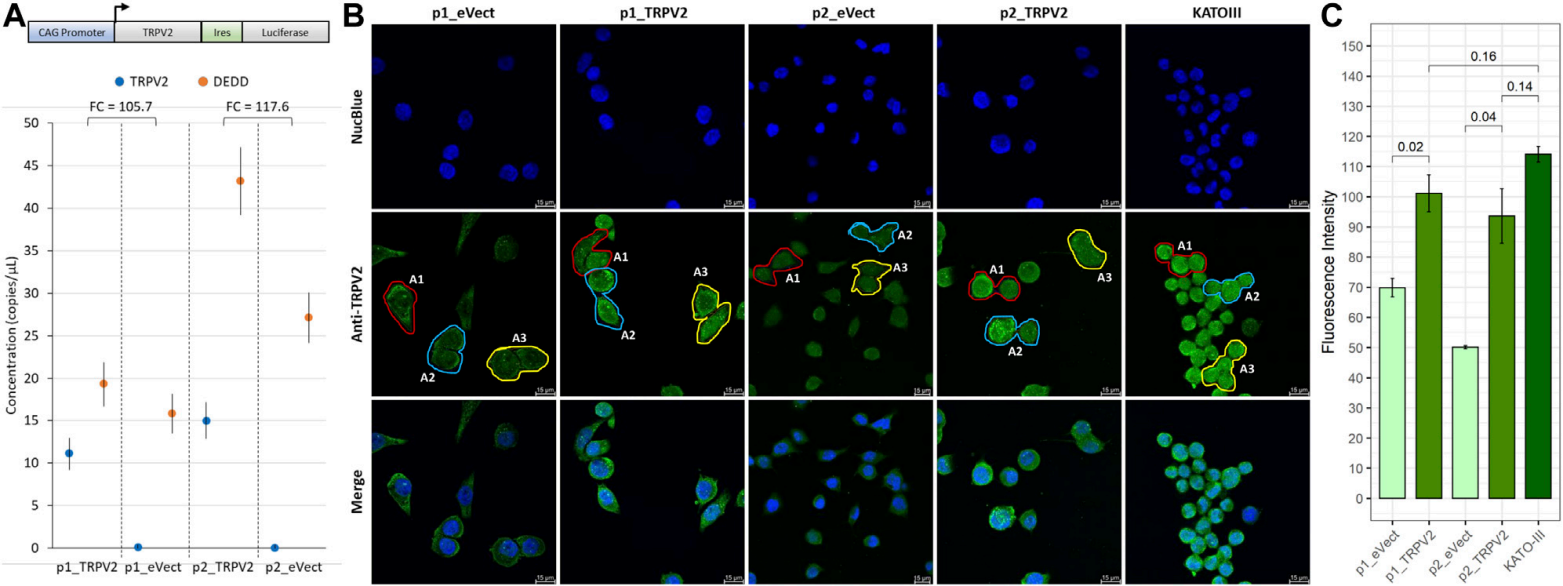
Exogenous expression of TRPV2 calcium channel in cisplatin-sensitive GC cell line. **(A)** Schematic representation of TRPV2 expression construct and evaluation of TRPV2 overexpression by ddPCR. **(B)** Representative images of TRPV2 exogenous expression by confocal microscopy in transformed AGS cell line pools as compared with the KATO-III cell line. **(C)** Quantification of TRPV2 expression in cells carrying the empty vector (p1_ and p2_eVect) or TRPV2 CDS (p1_ and p2_TRPV2) and comparison with KATO-III as reference. *t*-test on mean fluorescence intensity from three areas (A1, A2, and A3) was used for comparisons. Confocal specimen staining: NucBlue, rabbit anti-human TRPV2 revealed with goat anti-rabbit IgG-FITC; magnification: ×63; Leica SP8 microscope.

To confirm the association of TRPV2 activity with the gain of resistance to cisplatin-induced apoptosis, we overexpressed TRPV2 in a GC cell line sensitive to cisplatin, using the vector reported in Figure 5A. To this end, we engineered the AGS cell line that, as demonstrated above, was characterized by the lowest TRPV2 expression and the highest cisplatin sensitivity (Figure 2). Through G418 selection, we generated two pools of TRPV2 expressing clones (p1_TRPV2 and p2_TRPV2) and two pools of clones carrying the empty vector (p1_eVect and p2_eVect). Subsequently, we confirmed TRPV2 protein overexpression in the two pools by ddPCR and confocal microscopy. As shown in Figure 5A, the transformed pools (p1_TRPV2 and p2_TRPV2) expressed TRPV2 mRNA at levels over 100 times higher as compared with respective eVect control. TRPV2 overexpression was confirmed at protein level by confocal microscopy (Figure 5B). The expression pattern appears similar to that of the KATO-III cell line, which constitutively expresses TRPV2 at a high level, a feature that we think to be involved in its cisplatin resistance. Mean fluorescence intensity between TRPV2 expressing pools and eVect controls was significantly different (p1_TRPV2: 101.9 ± 6.1 vs. p1_eVect: 69.8 ± 3.1 *p* = 0.02; p2_TRPV2: 93.6 ± 9.0 vs. p2_eVect: 50.1 ± 0.56 *p* = 0.02) (Figure 5C). Data were obtained from three similar areas for each cell type (p1_eVect: 639 ± 9 µm^2^ vs. p2_eVect: 602 ± 9 µm^2^ vs. p1_TRPV2: 631 ± 33 µm^2^ vs. p2_TRPV2: 541 ± 28 µm^2^ vs. KATO-III: 530 ± 28 µm^2^, *p* = ns) on images acquired with the same laser excitation intensity and CCD camera settings.

**FIGURE 6:**
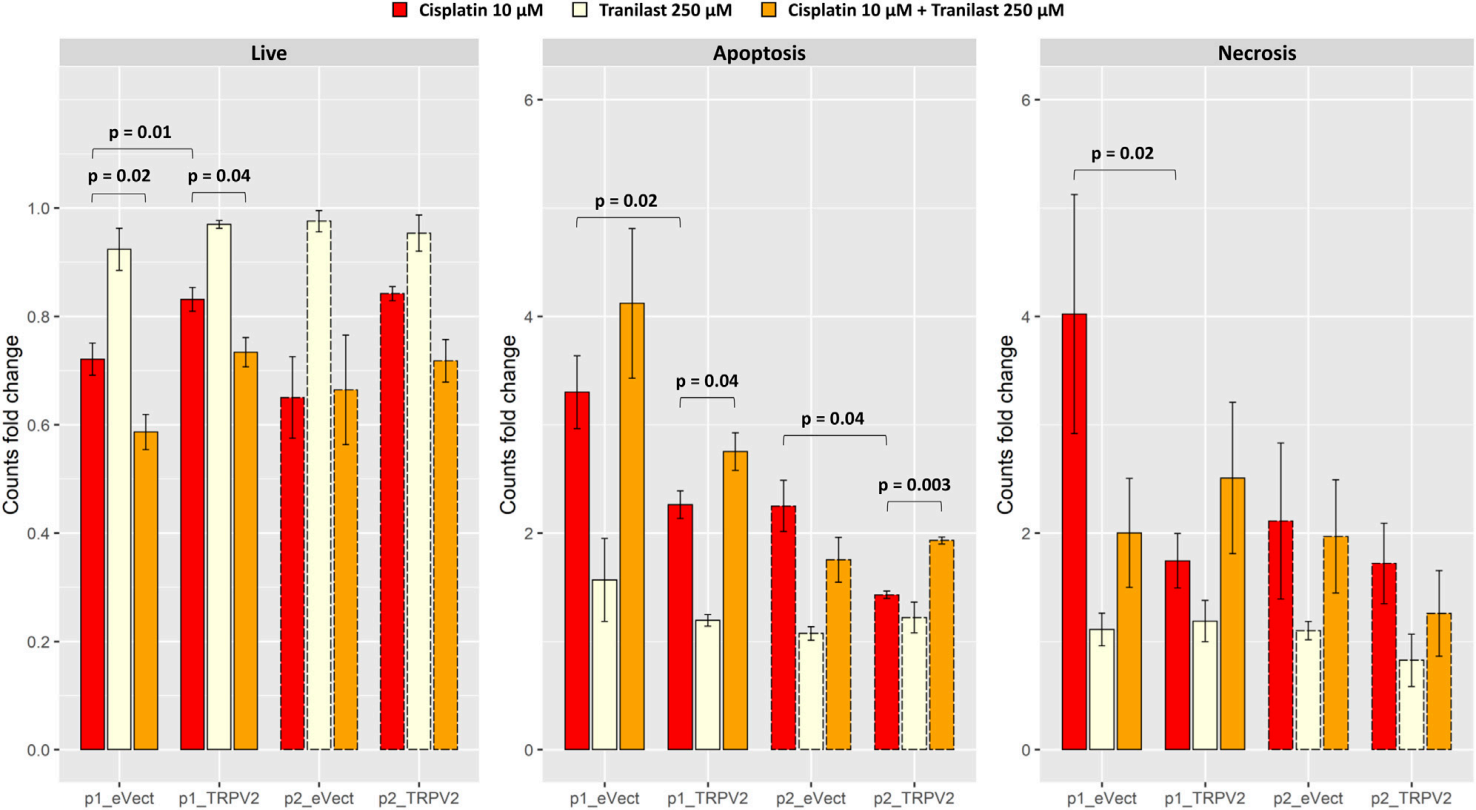
Effect of exogenous expression of TRPV2 calcium channel in the cisplatin-sensitive AGS GC cell line. Cisplatin-induced apoptosis in transformed AGS cell line pools expressing exogenous TRPV2 was assessed by flow cytometry. *t*-test was used to assess the significance of comparison between mean fold changes from three replicates (solid and dashed lines distinguish between the first and second pool).

After exposure of TRPV2 expressing pools to cisplatin, we observed the acquisition of a cisplatin-induced apoptosis resistant phenotype as compared with their controls (Figure 6). In particular, as compared with pools carrying empty vector, exogenous expression of TRPV2 determined an increase of viable cells after cisplatin treatment of about 20% (pool1 = +15% *p* = 0.01; pool2 = +30%). Notably, a significant reduction of live cells in p1_eVect and p1_TRPV2 were observed when tranilast was added to cisplatin as compared with cisplatin alone. Unlike for that observed in KATO-III cells, tranilast as a single agent did not influence cell viability.

Regarding cell death by apoptosis, overexpression of TRPV2 reduced apoptotic cells rates after exposure to cisplatin of over 30% (p1_TRPV2 vs. p1_eVect: −31%, *p* = 0.02; p2_TRPV2 vs. p2_eVect: −36%, *p* = 0.04). According to results of KATO-III cells (Figure 4B), the addition of tranilast to cisplatin significantly increased apoptosis rates, restoring the sensitivity to cisplatin-induced apoptosis in both pools of cell clones expressing exogenous TRPV2 (p1_TRPV2: +22%, *p* = 0.04; p2_TRPV2: +35%, *p* = 0.003). Notably, although there were some differences in the extent of apoptosis rates, the two TRPV2 expressing pools showed the same behavior. Moreover, the tranilast-mediated resistant phenotype reversion suggests a specific activity of this inhibitor on the TRPV2 channel.

No significant differences were observed for necrosis rates, except for the comparison between p1_eVect and p1_TRPV2 pools treated with cisplatin alone. In particular, TRPV2 exogenous expression reduced necrotic cell amount of about 57% (*p* = 0.02), another sign of TRPV2 role in resistance to cisplatin-induced cell death.

## Discussion

Several reports showed that TRPV2 is involved in the regulation of cell death or cancer migration/invasion in cancer cells (Liu and Wang, 2013; Liberati et al., 2014a; Zhou et al., 2014; Elbaz et al., 2018; Santoni and Amantini, 2019). TRPV2 may affect cancer biology through the regulation of Ca^2+^ signaling. Indeed, Ca^2+^ is the most abundant second messenger in humans, and it plays a role in the regulation of several physiological cellular processes that are commonly altered in cancer cells (Berridge et al., 2000; Xu et al., 2018). Intracellular Ca^2+^ concentration is maintained at very low levels. Its increments can act locally in so-called microdomains or propagate throughout the cytoplasm and elicit different responses depending on the involved cellular area (Csordás et al., 2010; Parkash and Asotra, 2010). Moreover, prolonged intracellular Ca^2+^ increase is toxic and drive cell death by apoptosis, a mechanism of notable relevance for cancer chemotherapy success and drug resistance (Monteith et al., 2007; Danese et al., 2017). Indeed, disruption of normal calcium homeostasis through efflux of calcium from the mitochondria is one of the cisplatin-mediated cell death mechanisms (Dasari and Tchounwou, 2014).

In the present study, we evaluated the possibility to consider TRPV2 as a candidate for targeted therapy since it is expressed at high levels in GC samples (Zoppoli et al., 2019) and it is relatively simple to target by small molecules due to its localization on the cell surface. Firstly, we demonstrated that GC samples with high expression of TRPV2 are characterized by a modulation of apoptotic process as compared with low TRPV2 expression ones (Figure 1). In particular, several antiapoptotic genes resulted in upregulation, such as BCL2, IGF1, SERPINE1, and IL6 (Pavón et al., 2015; Wei et al., 2001; Lin et al., 2001; Alexia et al., 2004). In *in vitro*, we observed a direct correlation of TRPV2 expression with resistance to cisplatin-induced apoptosis (Figure 2). Then, due to its outward rectification current (Caterina et al., 1999), we hypothesized that TRPV2 can confer cisplatin resistance through the efflux of Ca^2+^ ions, preventing its cytosolic accumulation and hence apoptosis activation. Accordingly, using ionomycin as an intracellular Ca^2+^ concentration inducer, we measured an increase of cytosolic Ca^2+^ in the presence of tranilast, a selective inhibitor of TRPV2 activity (Figure 3). Consequently, we demonstrated that tranilast, in combination with cisplatin, is capable to “unlock” apoptosis in cisplatin-resistant cells (Figure 4). Lastly, we evaluated the effect of TRPV2 overexpression in a GC cell line characterized by the highest apoptosis rates after cisplatin exposure. Notably, transformed cells acquired a cisplatin-resistant phenotype as compared with their controls carrying the empty vector (Figure 6). These results clearly show that TRPV2 promotes resistance to cisplatin-induced apoptosis in GC cells *via* the modulation of cytosolic Ca^2+^ levels. Although the present study lacks data on patient’s samples, based on our previous work, we are confident on the clinical translation of our results. Indeed, we reported that TRPV2 expression in GC patients is associated with worse prognosis, advanced pathologic stages, and, at protein level, it is expressed only in GC samples and not in normal gastric mucosa (Zoppoli et al., 2019).

The use of tranilast, initially developed as an antiallergic drug, was reported effective to inhibit the growth of various types of cancer (Darakhshan and Pour, 2015). A successful combination treatment with tranilast and cisplatin was tested on a xenograft model of scirrhous gastric cancer, in which a decrease of tumor size and an increase of apoptosis were reported (Murahashi et al., 1998). Moreover, it was reported that TRPV2 was overexpressed in leukemic blast cells compared to normal human blood cells, and TRPV2 silencing or pharmacological targeting by tranilast or SKF96365 triggered caspase-mediated apoptosis (Siveen et al., 2019). TRPV2 inhibition by tranilast was also cytotoxic and effectively decreased the number of tumor spheres in esophageal squamous cell carcinoma (Shiozaki et al., 2018). Consistent with our data, tranilast was proved effective to enhance the response to chemotherapeutic drugs in osteosarcoma cells in a dose-dependent manner, showing a synergistic effect with cisplatin and doxorubicin. In particular, consistent with our data, tranilast alone did not induce significant apoptosis, but the combination of tranilast with cisplatin induced early and late apoptotic cell death (Nakashima et al., 2019). Moreover, it was shown that, in a breast cancer mouse model, tranilast treatment upregulates p53 and induces PARP and CASP3 cleavage in *in vitro*, consistent with the promotion of tumor cell apoptosis (Subramaniam et al., 2010). In a recent work, through a synthetic lethality approach, the authors demonstrated that tranilast affects the expression of genes involved in several pathways that confer tolerance to cisplatin, including key platinum-transporting proteins such as ATOX1 (Mariniello et al., 2020). In this regard, since Ca^2+^ acts as a second messenger in several cell processes, gene expression modulation could reflect a TRPV2-mediated regulation of intracellular Ca^2+^ levels (De, 2011). Indeed, pathway analysis revealed that TRPV2 regulates malignant potentials of esophageal squamous cell carcinoma cells *via* cross-talk between the hedgehog pathway and Wnt/β-catenin signaling (Kudou et al., 2019). A contrasting role was described for TRPV2 overexpression in liver cancer in which its activity inhibited spheroid and colony formation and reduced stem cell markers expression in HepG2 cells. Indeed, blocking TRPV2 with tranilast, increased *in vitro* spheroid and colony formation and stem cell marker expression in liver cancer cell lines (Hu et al., 2018). Similarly, TRPV2 silencing promotes glioma cell survival and proliferation and resistance to Fas-induced apoptotic cell death in an ERK-dependent manner (Nabissi et al., 2010). However, this dual behavior of TRPV2 is not uncommon among cancer-associated genes, an example is TGFβ that can act as a tumor suppressor or tumor promoter according to the tumor stage (Cantelli et al., 2017).

In conclusion, although our study deserves further confirmation by *in vivo* studies and by clinical trials, we demonstrated the possible mechanism by which the pharmacological targeting of TRPV2 with tranilast can be useful to overcome drug resistance, a major limitation of current therapeutic regimens for GC. The main advantage of using tranilast in combination with other chemotherapeutics is that it is well tolerated, with no serious adverse events reported and, since it is long-lastingly used, detailed pharmacological data are available (Darakhshan and Pour, 2015).

## Data Availability

The TCGA-STAD dataset analyzed in this study can be found in The Cancer Genome Atlas repository (https://www.cancer.gov/tcga). All other data generated and analyzed in this study are included in the article.
